# Tiny infarction of rostral cerebellum manifested by contralesional body lateropulsion

**DOI:** 10.1016/j.ensci.2024.100523

**Published:** 2024-08-30

**Authors:** Obay Alalousi, Mickael Bonnan

**Affiliations:** a Department of Neurology, Delafontaine Hospital, 2, rue du Docteur Delafontaine, 93200 Saint-Denis, France; b Sorbonne Université, Faculté de médecine, 91-105 boulevard de l'hôpital, 75013 Paris, France

**Keywords:** Contralesional body lateropulsion, Paravermis lobule area, Spinocerebellar tracts, Cerebellar nuclei, Vestibular nuclei, Superior cerebellar peduncle

## Abstract

Body lateropulsion (BLP) has been reported several times after cerebellar infarction. It is usually ipsilateral to the cerebellar infarction, particularly when limited to the rostral cerebellum. In contrast, contralesional BLP after cerebellar infarction has been reported in more caudal regions of the cerebellum (such as the nodulus or the tonsil).

We report the case of a small infarction of the left anterior paravermis of the rostral cerebellum which resulted in bilateral symptoms: ipsilesional limb ataxia and, unexpectedly, contralesional BLP.

Several neurological pathways were potentially involved. Both right and left dorsal spinocerebellar tracts may have been damaged by the infarction of the left anterior paravermis. On the other hand, the proximity of the infarct to the superior cerebellar peduncle may have caused damage to the vestibular pathways (fastigio-vestibular or dentato-vestibular tracts), as they exit the cerebellum by the superior cerebellar peduncle. A lesion of the cerebellum close to the superior cerebellar peduncle could result in a contralesional BLP.

## Highlights

• Lesion in rostral cerebellum responsible of contralesional body lateropulsion.
• Vestibular pathways could be damaged by paravermal infarction.
• Early and complete recovery of body lateropulsion after paravermal infarction.

## 1 Introduction

Body lateropulsion (BLP) is a deficit of body orientation with respect to gravity in the frontal plane [1]. The body tilts to one side, increasing the risk of falling. BLP has been reported mainly after infarction of the medulla oblongata but also after infarction of the pons, the thalamus, around the red nucleus and in various locations of the cerebellum [2]. Cerebellar involvement is only observed in 15 % of cases [2]. BLP after infarction of the rostral cerebellum gives rise to ipsilateral BLP (iBLP) [3].

We present the case of a small infarction of the rostral cerebellum that unexpectedly resulted in contralesional BLP (cBLP) and ipsilesional limb ataxia. Potentially damaged neurological pathways are discussed.

## 2 Case history

A 70-year-old right-handed man with no medical history reported the sudden onset of gait instability, dizziness, and nausea. Minimal dysarthria was transient.

Clinical examination revealed permanent BLP on the right side when sitting, standing, and walking (Fig. 2). He had gait instability with a tendency to fall to the right when walking. When standing upright, he only leaned to the right but without the tendency to fall. He also displayed minor left lower limb ataxia on the heel-to-shin test. He had no ocular deviation or nystagmus. Dizziness and BLP disappeared over the following days.

The patient did not seem to be aware of his new postural condition. He had no rheumatological or orthopedic comorbidities.

**Fig. 1:**
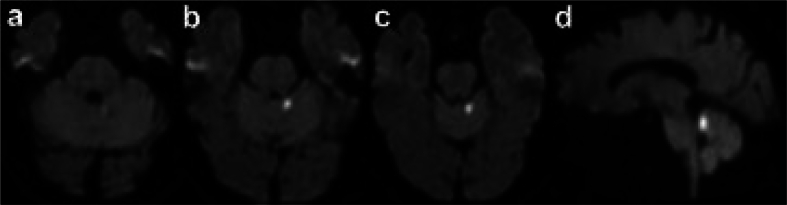
**Successive axial (a-c) and sagittal (d) brain MR images.** Focal ischemic lesion of the left anterior paravermis, just posterior and superior to the superior cerebellar peduncle (diffusion-weighted MRI sequences).

Brain MRI (Fig. 1) showed a small cerebellar ischemic stroke in the territory of the left superior cerebellar artery.

**Fig. 2:**
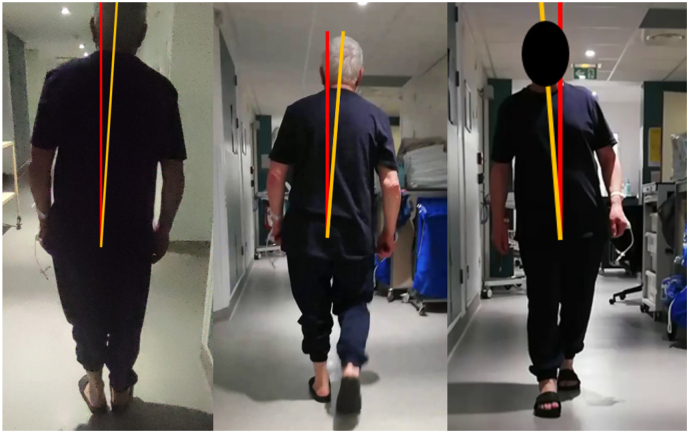
**Body lateropulsion.** BLP to the right side when standing (left), walking (middle and right), and sitting (not shown). Approximate anatomical axis of trunk (yellow) is shown in relation to vertical axis (red). (For interpretation of the references to colour in this figure legend, the reader is referred to the web version of this article.)

**Fig. 3:**
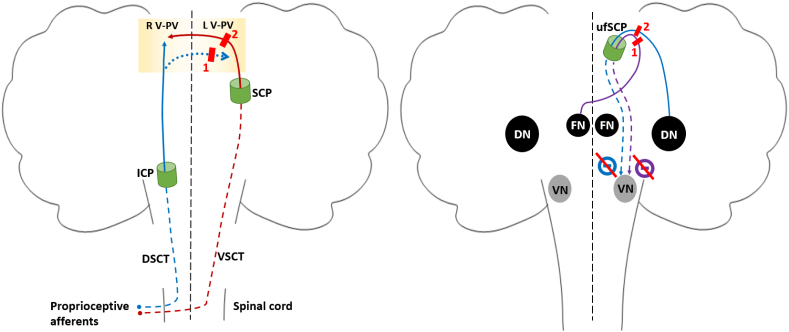
**Potentially damaged pathways.** **Left panel. Spinocerebellar tracts (SCT).** Interruption of the SCT in the left rostral cerebellum could lead to right cBLP, either by the interruption of inconstant crossed DSCT fibers (dotted blue line) after crossing the midline in the rostral cerebellum (1) or by the interruption of VSCT fibers (continuous red line) before recrossing the midline in the rostral cerebellum (2). **Right panel. Cerebello-vestibular pathways.** Crossed fastigio-vestibular and uncrossed dentato-vestibular fibers exert an inhibitory tone on the left vestibular complex, which is lost by the interruption of crossed fastigio-vestibular (1) or uncrossed dentato-vestibular fibers (2) by the cerebellar infarction, leading to a right BLP. Abbreviations: DN: dentate nucleus; DSCT: dorsal spinocerebellar tract; FN: fastigial nucleus; ICP: inferior cerebellar peduncle; L(R) V-PV: left(right) rostral vermis and anterior paravermis; SCP: superior cerebellar peduncle; ufSCP: uncinate fasciculus of the SCP; VN: vestibular nuclei complex in brainstem; VSCT: ventral spinocerebellar tract.

Repeated cardiac rhythm monitoring, transthoracic cardiac ultrasound, cervical and brain CT angiography were all normal.

## 3 Discussion

Post-stroke body lateropulsion is due to cerebellar damage in only 15 % of cases, whereas medullar stroke accounts for 59 % of BLP cases [2].

Our patient had a small infarct confined to the left anterior paravermis, just posterior and superior to the superior cerebellar peduncle (SCP) resulting in an unexpected contralateral BLP.

The direction of BLP (iBLP or cBLP) after a cerebellar lesion depends on its anatomic site. BLP after rostral cerebellar infarction has been reported to be ipsilesional [3], whereas cBLP has been described in more caudal cerebellar lesions, particularly in the nodulus or the tonsil [4].

To our knowledge, a tiny, isolated infarction localized specifically to the anterior paravermis has never been reported in association with cBLP. This exceptional contralateral clinical pattern may be caused by damage affecting pathways passing through the rostral cerebellum.

The spinocerebellar tracts mainly project to the rostral vermis and anterior paravermis [5] and are mainly of two types: dorsal spinocerebellar tract (DSCT) and ventral spinocerebellar tract (VSCT) (Fig. 3, left).

A lesion specifically affecting the DSCT (whether in the spinal cord, in the medulla oblongata or at its termination in the rostral vermis) has been incriminated in several cases of iBLP [6,7]. Indeed, the DSCT, which remains ipsilateral throughout its path, conveys non-conscious proprioceptive information from the ipsilateral lower extremities and trunk to the ipsilateral rostral vermis and anterior paravermis [5]. However, tractography of the DSCT showed that it crosses the midline in 48 % of cases reaching the contralateral areas [8]. Thus, the left anterior paravermis stroke in our case could have damaged both the right crossed DSCT (manifested by right BLP) and the left uncrossed DSCT (manifested by left lower limb ataxia). However, DSCT interruption has been shown to affect BLP mainly in gait [7], whereas our patient had BLP in both static and dynamic situations.

VSCT also conveys non-conscious proprioceptive information from body to cerebellum and decussates twice: at the spinal cord entrance and in the rostral cerebellum through the contralateral SCP, to terminate in the ipsilateral rostral vermis and anterior paravermis [5]. Lesion of the VSCT before recrossing the midline might explain the cBLP in our patient. However, VSCT roles are incompletely understood. VSCT mainly convey the motor information generated by the spinal cord to the cerebellum and help to modulate the contralateral motor response at the level of the spinal interneurons [5,9]. Whether VSCT involvement could alter posture in our patient would require further investigation.

On the other hand, our patient initially presented with vertigo and nausea and was unaware of his BLP. Thus, a misperception of verticality is suspected, and vestibular tone imbalance might have been involved. The subjective visual vertical (SVV) test, which is around 0° in healthy subject in an upright position, is used to investigate vestibular tone imbalance in the frontal plane. Lesions of the SCP are associated with contraversive tilts on the SVV test [10], supporting its role in the perception of verticality. In our patient, the lesion was close to the left SCP, suggesting that the two cerebello-vestibular pathways exiting the cerebellum through the SCP (fastigio-vestibular and dentato-vestibular pathways) could have been damaged (Fig. 3, right).

The fastigial and dentate nuclei are deep cerebellar nuclei connected with the vestibular nuclei complex (VN) in the brainstem [11,12].

The fastigial nucleus (FN) exerts an overall inhibitory tone on the contralateral VN [13]. FN fibers to the contralateral VN cross the midline in the cerebellum, then leave it through the contralateral SCP via the uncinate fasciculus while FN fibers to the ipsilateral VN leave the cerebellum by the ICP, without crossing the midline [12].

A lesioned dentate nucleus (DN) was shown to be responsible for a contraversive tilt on the SVV test and therefore a disturbance of the internal pattern of verticality [10,14]. The DN is thought to exert an inhibitory effect on the ipsilateral VN [14].

Cerebellar infarction in our patient was located close to the left SCP. The crossed fastigio-vestibular fibers or the uncrossed dentato-vestibular fibers, both exerting an inhibitory tone on the left VN, could have been damaged just before entering the left SCP (Fig. 3, right). This may have resulted in an increased tonic activity of the left VN leading to right SVV tilt and right BLP.

In conclusion, a tiny lesion of the rostral cerebellum around the junction with the SCP may result in cBLP, although the responsible pathway remains putative.

## Ethics

Written consent was obtained for the publication of the anonymized figures.

## Funding

This report did not receive any specific grant from funding agencies in the public, commercial, or not-for-profit sectors.

## CRediT authorship contribution statement

**Obay Alalousi:** Writing – review & editing, Writing – original draft, Formal analysis, Conceptualization. **Mickael Bonnan:** Writing – review & editing, Supervision, Formal analysis.

## Declaration of competing interest

None.
